# Supplementary material for: Transcriptomic Insights into Mechanisms of Early Seed Maturation in the Garden Pea (Pisum sativum L.)
Source: Cells. 2020 Mar 23;9(3):779. doi: 10.3390/cells9030779 (PMC7140803; doi:10.3390/cells9030779)

Zhewan−1(10) vs Zhew

Global Summary

%DE = 0.9

genes with fdr < 0.2 = 544 (190 + / 354 −)

genes with fdr < 0.1 = 522 (177 + / 345 −)

genes with fdr < 0.05 = 475 (155 + / 320 −)

genes with fdr < 0.01 = 424 (138 + / 286 −)

genes in genesets = 0

<FC> = 0

<t−score> = 70233.8

<p−value> = 0

<fdr> = 0.1

Portrait

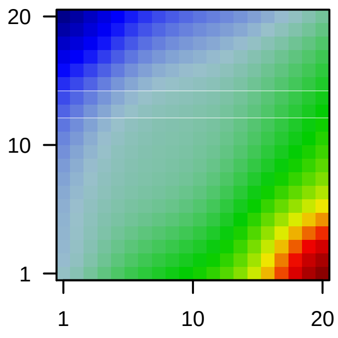

Top 100 DE genes

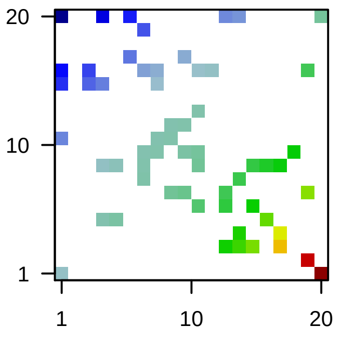

Global Genelist

Rank	ID	log(FC)	p−value	fdr	Metagene	Description
1	TRINITY_DN1	15.24	0e+00	6e−09	20 x 1	
2	TRINITY_DN2	1.51	0e+00	6e−09	20 x 1	
3	TRINITY_DN7	−0.84	0e+00	6e−09	20 x 20	
4	TRINITY_DN6	0.69	0e+00	6e−09	20 x 20	
5	TRINITY_DN1	−2.18	2e−10	6e−09	1 x 20	
6	TRINITY_DN3	−18.92	2e−10	2e−08	1 x 20	
7	TRINITY_DN6	0.5	9e−10	2e−08	19 x 7	
8	TRINITY_DN7	0.18	9e−10	2e−08	15 x 9	
9	TRINITY_DN1	−5.79	9e−10	1e−07	1 x 1	
10	TRINITY_DN6	−0.27	3e−09	1e−07	14 x 20	
11	TRINITY_DN1	2.82	4e−09	1e−07	20 x 1	
12	TRINITY_DN1	0.06	5e−09	3e−07	5 x 5	
13	TRINITY_DN1	0.24	1e−08	3e−07	14 x 3	
14	TRINITY_DN2	4.15	1e−08	3e−07	20 x 1	
15	TRINITY_DN5	−0.26	2e−08	3e−07	7 x 19	
16	TRINITY_DN3	0.2	3e−08	4e−07	14 x 8	
17	TRINITY_DN3	−0.12	3e−08	4e−07	8 x 16	
18	TRINITY_DN3	−0.31	4e−08	4e−07	1 x 15	
19	comp15249_c	0.28	4e−08	4e−07	18 x 10	
20	TRINITY_DN6	0.3	5e−08	4e−07	16 x 9	

p−values

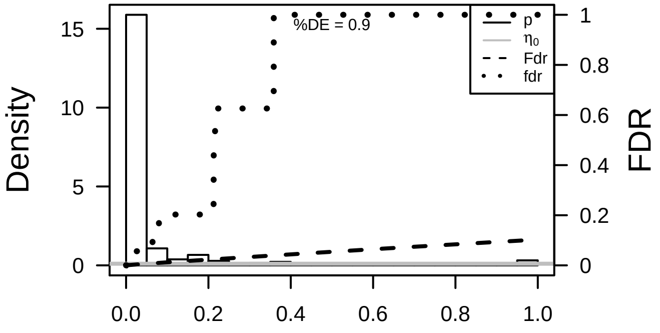

Supplement: Supplementary file 1 [file cells-09-00779-s001.zip › Supplementary materials/FileS3.zip!/FileS2/Hormones - Results/Summary Sheets - Differences/Reports/Zhewan.1.10..vs.Zhewan.1.25..pdf]
